# Early identification of struggling learners: using prematriculation and early academic performance data

**DOI:** 10.1007/s40037-019-00539-2

**Published:** 2019-09-27

**Authors:** Layne D. Bennion, Dario Torre, Steven J. Durning, David Mears, Deanna Schreiber-Gregory, Jessica T Servey, David F. Cruess, Michelle Yoon, Ting Dong

**Affiliations:** 1 Uniformed Services University of Health Sciences, 20814 Bethesda, MD USA; 2 School of Medicine, University of Colorado, Aurora, CO USA

**Keywords:** Medical education, Remediation, Identification of struggling learners

## Abstract

**Introduction:**

A perennial difficultly for remediation programmes in medical school is early identification of struggling learners so that resources and assistance can be applied as quickly as is practical. Our study investigated if early academic performance has predictive validity above and beyond pre-matriculation variables.

**Methods:**

Using three cohorts of medical students, we used logistic regression modelling and negative binomial regression modelling to assess the strength of the relationships between measures of early academic performance and outcomes—later referral to the academic review and performance committee and total module score.

**Results:**

We found performance on National Board of Medical Examiners (NBME) exams at approximately 5 months into the pre-clerkship curriculum was predictive of any referral as well as the total number of referrals to an academic review and performance committee during medical school (MS)1, MS2, MS3 and/or MS4 years.

**Discussion:**

NBME exams early in the curriculum may be an additional tool for early identification of struggling learners.

## What this paper adds

This original research addresses the problem of identification of academically struggling medical students within the first several months of pre-clerkship curriculum using a combination of pre-matriculation data and academic performance on early exams. This combination allows for early application of remediation resources, potentially reducing costs of remediation.

## Introduction

The need for academic remediation is significant given 10% of medical students encounter an academic failure at some point during their training [1]. Unfortunately, studies also suggest that the later into the curriculum a struggling learner is identified, the higher the likelihood that s/he will present with exhaustion and discouragement, requiring more extensive make-up, which will potentially have a negative impact on the student’s ability to modify or make changes in their study process [2–4]. While it is possible there may be some unintended consequences to early identification of individual at-risk students (e.g. stigma, false positives), it also enables prompt remediation efforts. Efforts early in a struggling student’s process are thought to be less costly to the system and enable the struggling student to receive needed resources and accelerate their adaptation.

Some contributors for at-risk medical students are well known such as gender, ethnic minority status, undergraduate grade point average (GPA) and Medical College Admissions Test (MCAT) scores [5–7]. More recently several investigations have focused on whether performance measures prior to matriculating in medical school predict struggling learners. For example, Woolf et al. [6] found, in their meta-analysis of UK physician training, that undergraduate GPA predicted 23% of the variance in medical school grades. Saguil and colleagues [8] found weak correlations between MCAT scores and medical school grades and moderate correlations between MCAT scores and later performance on National Board of Medical Examiners (NBME) step examinations [8]. Donnon and associates’ [7] meta-analysis of MCAT score’s predictive validity of later medical school performance found small to medium effect sizes across various academic performance measures. These contributors, however, predict a relatively small portion of the variance in academic performance and thus are not particularly useful in the early identification of struggling learners. Further, the identification of markers of struggling learners early in medical school is lacking. To our knowledge, no studies of medical students have attempted to identify struggling learners early on in medical school by combining pre-matriculation variables with early academic performance data to predict subsequent performance during medical school.

A typical process identifying an academically at-risk medical student is through a formal process led either by individual faculty or a competency review committee [9]. Typically, such committees review a student’s record after academic failure(s) or other performance problems (e.g. professionalism) and then make formal recommendations to the school leadership regarding that particular student’s status within the school. While there is little in the published literature about these committees and their processes [10], in our experience these reviews occur after an academic failure and/or several suboptimal performances. These institutional-level processes are perceived as very punitive by some students [10], and are designed to capture those students with the most serious academic or professional behaviour problems (i.e. those who are at risk of being disenrolled).

Ideally, at-risk students are identified within the first several months of pre-clerkship work and prior to the formal institutional processes such as competency committee identification. If prompt identification occurs, then remediation services can be applied early. Stegers-Jager and colleagues [4] presented a proactive identification and engagement model within a Dutch medical school utilising markers of substandard progress through their programme at months 4, 7 and 12. Those students are then offered voluntary engagement with a support programme prior to any formal designation of academic probation. Their work found that at-risk students who engaged in the early voluntary support programme completed the first-year curriculum at a significantly higher rate than those who did not participate.

Therefore, the purpose of this study was to investigate the association between the combination of common pre-matriculation and academic performance measures in the first 5 months of medical school with later referral to the student promotion committee (5 months equates to the first two curriculum modules at our institution). We had three overarching research questions. First, is academic performance in the first 5 months of medical school associated with the risk of formal referral to the academic performance review committee later in medical school? Second, does academic performance in the early pre-clerkship modules correlate with the total number of referrals to an academic performance review committee later in medical school? Third, are early pre-clerkship module performance measures associated with committee referrals? We hypothesised that poorer academic performance relative to peers with similar pre-matriculation performance early on in medical school would be associated with a higher chance of getting formal referral to the academic performance committee as well as being associated with a higher frequency of such referrals.

## Methods

### Sample and study context

This study is part of the Long-term Career Outcome Study conducted at the F. Edward Hebért School of Medicine, Uniformed Services University of the Health Sciences (USUHS). USUHS matriculates approximately 170 medical students annually. The study sample is the School of Medicine graduating classes of 2015–2017 (*n* = 522). This study was approved by the USUHS Institutional Review Board (file number MED-83-9698).

### Study measures

#### Pre-matriculation measures

We included commonly obtained measures—gender, age, MCAT score and undergraduate GPA. Given Dyrbye and associates’ [11] finding that having children is associated with lower levels of medical student depressive symptoms, which can affect academic performance, we also included the number of family members a student has primary legal or financial responsibilities for at matriculation. These pre-matriculation measures were used as the baseline or control variables in the study. Other pre-matriculation scores, such as selection ratings or interview ratings, were not available.

#### Performance measures in the first 5 pre-clerkship months

We looked specifically at academic performance in the first two pre-clerkship modules: Foundation in Medicine (Foundation) and Musculoskeletal Integument (MSK). These modules are 7‑ to 9‑week periods that focus on particular themes or organ systems and integrate interrelated basic and clinical science concepts and knowledge. We used students’ NBME exam performance, midterm and final NBME exams, from these two modules as the primary explanatory variables in the study as the NBME exam scores would be most applicable to other medical schools. In addition, a measure of overall performance from each module was also utilised. This measure, average module score, includes NBME scores as well as student performance on numerous other assessments. While the NBME exam performance is integrated into the average module score (includes all assessments for that module), each model separately considered the predictive strength of each performance variable. These pre-clerkship measures (mid-and final-module NBME exam scores and overall module performance scores) were the primary explanatory variables of interest.

The NBME assessments are purchased from the NBME Customised Assessment Service (CAS). Each exam consists of 80–100 questions from the CAS’s pool of available items, selected by the content experts who teach or oversee the module. Faculty-developed (FD) exams are also included as a part of each module. These exam questions typically cover content for which appropriate questions cannot be found in the CAS item pool. At the end of the module, each student’s overall module average is calculated using the NBME and FD exam scores, as well as other numerically scored assessments, such as quizzes, practical exams and small group exercises (e.g. anatomy lab). Module letter grades are determined based on the overall module average score as well as considering any below passing assessments within the module.

#### Outcome measures

We included two outcome measures in the study: (1) whether a student was referred to the Student Promotions Committee (SPC) and (2) how many times a student was referred to the SPC during the later portion of medical school (MS)1 or during MS2, MS3 and/or MS4 years. The SPC is the academic performance review committee at USUHS. The SPC comprises ten or more medical school faculty from a variety of clinical and basic science disciplines who review the entire performance record of all referred students and then make formal recommendations to the school’s leadership regarding that particular student’s status within the school [12].

In order to minimise any chance that results might be impacted by a direct relationship between the predictor (NBME scores in the first two modules) and the outcome variable (later referral to the SPC), students with poor overall performance in either of the first two modules (which resulted in referral to the SPC) were excluded from the analyses. This action resulted in removing 22 students (all three cohorts) from the analyses resulting in a final sample of 500 students.

### Statistical analysis

The statistical analysis consisted of three parts—descriptive statistics, logistic regression modelling and negative binomial regression modelling. For descriptive statistics, we reported means, standard deviations, and ranges for ratio variables and the frequency counts and valid percentage for nominal variables.

Multivariable logistic regression modelling was applied to examine the strength of associations between the explanatory variables and the first outcome variable, i.e. whether a student was ever referred to the SPC. For the second outcome variable of how many times a student was referred to the SPC, we performed negative binomial regression modelling because the distribution of this outcome variable was highly skewed (skewness = 2.85, kurtosis = 9.45) with a high portion of the values being zero (73.7%; zero meaning no referral). Negative binomial regression modelling provides a better model fit than Poisson regression model or ordinary least-squares regression model for this type of analysis.

For both the logistic and negative binomial regression modelling, students’ age, gender, undergraduate GPA, MCAT score and number of family members were entered as baseline (or control) variables. In the next step, students’ academic performance indicators from the first two modules (Foundations and MSK) were entered to examine their predictive power on the outcome variables above and beyond the baseline measures. Given one of our research goals was to examine which pre-clerkship academic performance indicators have a stronger association with the outcome variables and because the order of entering the explanatory variables in a regression model would have an impact on the results associated with a particular explanatory variable, we set up different regression models which included the pre-clerkship measures. For example, in model 1, after the demographic variables were entered, we entered Foundations module midterm NBME score; in model 2, we entered Foundations module final NBME score and so on. The various logistic regression models were compared on Nagelkerke *R*^*2*^. Nagelkerke *R*^*2*^ is commonly reported for logistic regression modelling. This statistic behaves like *R*^*2*^ in ordinary least-squares regression modelling. It has a range of 0 to 1 and a larger value indicates that a larger portion of the variance of the outcome variables is explained by the explanatory variables. The negative binomial regression models were compared on goodness of fit measures via Akaike information criteria (AIC) and Bayesian information criterion (BIC). For the regression coefficient estimates of the baseline (control) variables, we reported only those of model 1. As mentioned above, for both logistic regression modelling and negative binomial modelling, we tested one model at a time, which contains only one primary explanatory variable of interest in addition to the baseline measures. Thus, collinearity is not a concern. The purpose is to compare the predictive power of early poor academic performance on later SPC referral above and beyond the predictive power of pre-clerkship measures using the same set of baseline variables. All the statistical analyses were conducted in IBM SPSS 24.0.

## Results

### Descriptive statistics

Tab. 1 displays the means, standard deviations and ranges of the variables used. Given our students are similar to those of other US medical schools on the pre-matriculation variables, e.g. age, gender proportions, MCAT scores and undergraduate GPA, no additional analyses focused on these variables.

**Table 1:** Descriptive statistics of the measures of interest

*Variable on ratio scale*	*Mean*	*SD*	*Range*
Number of referrals to SPC	0.58	1.34	0–10
Age (at matriculation)	24.41	3.66	20–39
MCAT total score	31.14	2.48	25–41
Undergraduate overall GPA	3.57	0.26	2.55–4.00
Number of dependents	0.19	0.69	0–7
Foundation module midterm NBME score	74.94	8.38	48–95
Foundation module final NBME score	76.57	7.44	55–96
Module average score (average of all assessments during Foundation module)	80.06	7.28	62.44–96.64
MSK module midterm NBME score	79.92	7.03	56–99
MSK module final NBME score	83.57	7.40	57–100
Average module score (average of all assessments during MSK module)	82.20	5.08	63.88–92.52
*Variables on nominal scale*	*Category*	*Frequency*	*Valid percentage*
Referral to SPC	Yes	384	76.95
	No	115	23.05
Gender	Female	162	33.26
	Male	325	66.74
*Number of referrals*	*Number of students*	*Percentage*	
0	384	73.7	
1	33	6.3	
2	49	9.4	
3	20	3.8	
4	11	2.1	
5	10	1.9	
6–11	14	2.8	

*SPC* Student Promotions Committee, *MCAT* Medical College Admissions Test, *GPA* grade point average, *NBME* National Board of Medical Examiners, *Foundation* Foundation in Medicine, *MSK* Musculoskeletal Integument

### Logistic regression modelling

Tab. 2 displays the results of logistic regression modelling on the outcome variable of whether a student was ever referred to the SPC. The second module academic performance indicators were more strongly associated with referral to the SPC than the counterparts from the first module. Comparing the Nagelkerke *R*^*2*^ values associated with each model, the models show that the NBME final exam scores were more predictive than the overall module average score or the NBME midterm performance across both modules. The second module final NBME exam score was the most predictive variable [odds ratio = 0.90; 95% confidence interval (CI) = (0.87, 0.93); model Nagelkerke *R*^*2*^ = 0.22] of whether a student was referred to the SPC. Thus, given the same performance level (or value) for the baseline variables (gender, age, number of dependents, MCAT total score, undergraduate overall GPA), as students’ second module NBME final exam score decreased by 1 point, we saw an approximately 10% increase in the odds of that student being referred to the SPC at some point during the 4 years of medical school.

**Table 2:** Logistic regression modelling of the outcome variable of whether a student was referred to the SPC

Explanatory variable	Exp (B) (*p-*value)	95% CI of Exp (B)	Nagelkerke *R*^*2*^
*Pre-matriculation variables*			
Gender (male as reference group) (Model 1)	0.44 (*p* < 0.01)	(0.26, 0.76)	
Age (Model 1)	0.97 (*p* = 0.53)	(0.90, 1.06)	
Number of dependents (Model 1)	0.87 (*p* = 0.56)	(0.55, 1.38)	
MCAT total score (Model 1)	0.88 (*p* < 0.05)	(0.79, 0.98)	
Undergraduate overall GPA (Model 1)	0.96 (*p* < 0.01)	(0.97, 0.99)	
*Adding pre-clerkship performance variables*			
Model 1: Foundation module midterm NBME score	0.96 (*p* < 0.01)	(0.93, 0.99)	0.13
Model 2: Foundation module final NBME score	0.92 (*p* < 0.01)	(0.89, 0.95)	0.18
Model 3: Average module score (average of all assessments during Foundation module)	0.98 (*p* = 0.20)	(0.95, 1.01)	0.11
Model 4: MSK module midterm NBME score	0.90 (*p* < 0.01)	(0.87, 0.93)	0.21
Model 5: MSK module final NBME score	0.90 (*p* < 0.01)	(0.87, 0.93)	0.22
Model 6: Average module score (average of all assessments during MSK module)	0.92 (*p* *<* 0.05)	(0.86, 0.99)	0.13

*SPC* Student Promotions Committee, *MCAT* Medical College Admissions Test, *GPA* grade point average, *Foundation* Foundation in Medicine, *NBME* National Board of Medical Examiners, *MSK* Musculoskeletal Integument

### Negative binomial regression modelling

Tab. 3 showed the results of negative binomial regression modelling on the outcome variable of how many times a student was referred to the SPC. Similar to the results of logistic regression modelling, all the academic performance indicators during the module were significant predictors of this outcome measure. For the outcome variable of number of times referred to the SPC, the second module midterm NBME exam score showed the strongest relationship [Exp(B) = 0.91; 95% CI = (0.89, 0.94); AIC = 808.32; BIC = 837.20]. When the MSK midterm NMBE exam score was in the model, the exponential of the regression coefficient estimate was the smallest and the model fit was reflected best by the smallest values of both AIC and BIC. Thus given the same performance level for the baseline variables, as a student’s second module NBME midterm exam score decreased by 1 point, we saw their total number of referrals multiplicatively increase by a factor of 1.10 (1/0.91).

**Table 3:** Negative binomial regression modelling of the outcome variable of how many times a student was referred to the SPC

Explanatory variable	Exp (B) (*p-*value)	95% CI of Exp (B)	AIC, BIC
*Pre-matriculation variables*			
Gender (male as reference group) (Model 1)	0.62 (*p* < 0.05)	(0.43, 0.90)	
Age (Model 1)	1.00 (*p* = 0.89)	(0.94, 1.05)	
Number of dependents (Model 1)	0.73, (*p* = 0.10)	(0.50, 1.07)	
MCAT total score (Model 1)	0.91, (*p* < 0.05)	(0.84, 0.98)	
Undergraduate overall GPA (Model 1)	0.98, (*p* < 0.01)	(0.98, 0.99)	
*Adding pre-clerkship performance variables*			
Model 1: Foundation module midterm NBME score	0.96, (*p* < 0.01)	(0.94, 0.98)	(849.68; 878.57)
Model 2: Foundation module final NBME score	0.92, (*p* < 0.01)	(0.90, 0.95)	(823.46; 852.33)
Model 3: Module average score (average of all assessments during Foundation module)	0.97, (*p* < 0.01)	(0.95, 0.99)	(859.49; 888.36)
Model 4: MSK module midterm NBME score	0.91, (*p* < 0.01)	(0.89, 0.94)	(808.32; 837.20)
Model 5: MSK module final NBME score	0.91, (*p* < 0.01)	(0.89, 0.94)	(810.06; 838.93)
Model 6: Module average score (average of all assessments during MSK module)	0.92, (*p* < 0.01)	(0.87, 0.96)	(342.91; 364.73)

*SPC* Student Promotions Committee, *AIC* Akaike information criteria, *BIC* Bayesian information criterion, *MCAT* Medical College Admissions Test, *GPA* grade point average, *Foundation* Foundation in Medicine, *NBME* National Board of Medical Examiners, *MSK* Musculoskeletal Integument

## Discussion

This study aimed to investigate whether early academic performance variables predicted later referral to the SPC above and beyond common pre-matriculation measures. Because we excluded students who had very low performance on NBME exams during the first two modules, the study outcomes are independent of early poor academic performance directly leading to early referral to the SPC. Academic performance measures from both the first and the second pre-clerkship modules had a statistically significant association with both outcome variables (a student being referred to the SPC at any point as well as the total number of referrals to the SPC). While NBME exam results in general are predictive (minus the very first exam), the strongest single predictor (beyond that accounted for by pre-matriculation data) of the outcome variables was performance on the NBME midterm or final exams during the second module. These exams occur approximately 4–5 months into the pre-clerkship curriculum. Thus, it appears these early academic performance markers are important risk predictors of later academic performance problems.

Our study is the first we are aware of to evaluate the predictive validity of pre-matriculation information combined with early academic performance in medical school as reflected in referrals to the SPC later during the curriculum. Early intervention is important as it is hypothesised to prevent exhaustion and/or discouragement and could also lessen the impact of, or even prevent, formal action from the school.

Early identification of struggling learners is also important as it affords the chance for students to receive needed resources and guidance enabling course correction. Early identification of struggling learners is consistent with a model of remediation zones [9]. That is, early identification enables remedial action (zone 2), which at that stage in the curriculum is supportive, informal and short-term. Ideally such remedial action returns a student to normative learning pathways and prevents later formal remediation (zone 3), which involves more time, personnel and resources. While more research is needed regarding the costs, benefits and impacts of early versus later remediation efforts, it seems logical that early intervention would be less costly to the system and less draining for the student.

An additional application of using early academic performance might be in combination with portfolio approaches for student evaluation [13]. Portfolio approaches can enable recognition of subthreshold or subtle student performance patterns, which can escape notice in systems using only traditional grades. Combining subtle concerning patterns with other indicators of risk may further enable accurately identify struggling learners.

It is notable that the customised NBME examinations were predictive of future referral to the SPC. This is reassuring that faculty, empowered to build high-quality multiple-choice questionnaires, do so by targeting important fundamental concepts. Lack of mastery of these fundamental concepts eventually results in referral to the academic review committee. This association was not seen with other module scores, which may be due to less optimal statistics using FD examination materials, insufficient sampling with these other measures and/or range restriction for these measures in the pre-clerkship period.

We believe that our study provides encouraging news for academic leaders in terms of predicting which students may struggle during medical school. Given a percentage of students have a high frequency of repeat referrals to an academic performance committee, this suggests promise for early interventions in reducing the frequency of such referrals as well as reducing opportunity costs within medical schools challenged by at-risk students. Once at-risk students have been identified there are, of course, many individual factors to consider when designing individualised remediation plans, e.g. efficiency of study practices, efficiency of time management, personal stressors and self-care. Future studies should investigate if interventions targeting at-risk students who are identified earlier in the curriculum lessens prevent referrals to academic review and performance committees.

Our investigation had some limitations. This study was conducted at one single institution, which limits the generalisability of the study. However, the explanatory variable in our study, NBME exam scores, is commonly used across US medical schools. Second, this was a retrospective study from which we can infer only association not causation. Third, risk modelling is based on group trends and therefore does not provide calculated predictive values for each individual. Fourth, low scores on early NBME exam performance can be a contributing factor to an initial referral to the academic performance review committee (one exam score, by itself, is not sufficient for a referral); however, the outcome variable included all reviews across the entire 4 years of medical school for each student. Fifth, we did not have access to other pre-matriculation scores, such as selection ratings or interview ratings, which may have added additional predictive value.

Our study investigated the predictive validity of early pre-clerkship academic performance combined with pre-matriculation data in identification of struggling learners. In support of our first and third research question, we found that performance on NBME exams at approximately 5 months into the pre-clerkship curriculum does predict elevated risk of referral to the academic performance review committee later in medical school. In support of our second research question, performance on early NBME exams also predicted the number of referrals to the academic performance review committee. Our findings suggest that the use of customised NBME examinations early in the curriculum shows promise as an additional tool to identify struggling learners. This suggests that local faculty recognise the important concepts to master, and such early identification could allow more opportunities for corrective actions in struggling learners while reducing financial and other costs associated with remediation.
